# Green Chemistry and Multivariate Optimization in the Extraction of Phenolic Compounds: The Potential of NaDES in Alternative Raw Materials for Expanded Extrudates

**DOI:** 10.3390/mps8040082

**Published:** 2025-07-23

**Authors:** Mateus Alves Araújo, Bianca Rodrigues Morais, João Pedro da Silva Santos, Larissa Karla de Jesus, Kaliston Aurélio Lomba, Gustavo Costa do Nascimento, Marcus Alvarenga Soares, Nathalia de Andrade Neves, Irene Andressa, Maria Teresa Pedrosa Silva Clerici, Marcio Schmiele

**Affiliations:** 1 Institute of Science and Technology, Federal University of Jequitinhonha and Mucuri Valleys, Diamantina 39100-000, Minas Gerais, Brazil; mateus.araujo@ufvjm.edu.br (M.A.A.); bianca.morais@ufvjm.edu.br (B.R.M.); joao-pedro.santos@ufvjm.edu.br (J.P.d.S.S.); larissa.karla@ufvjm.edu.br (L.K.d.J.); kaliston.lomba@ict.ufvjm.edu.br (K.A.L.); nathalia.neves@ict.ufvjm.edu.br (N.d.A.N.); 2 School of Food Engineering, University of Campinas, Campinas 13083-970, São Paulo, Brazil; g155650@dac.unicamp.br (G.C.d.N.); mclerici@unicamp.br (M.T.P.S.C.); 3 Faculty of Agricultural Sciences, Federal University of Jequitinhonha and Mucuri Valleys, Diamantina 39100-000, Minas Gerais, Brazil; marcus.alvarenga@ufvjm.edu.br; 4 Departament of Food Technology, Federal University of Viçosa, Viçosa 36570-900, Minas Gerais, Brazil

**Keywords:** AGREE metric, extrusion, green extraction, legumes, cereal, snacks, multivariate analysis

## Abstract

Phenolic compounds are secondary metabolites widely distributed among plants, with bioactive properties, especially antioxidant activity. The search for sustainable extraction methods has driven the use of natural deep eutectic solvents (NaDESs), formed by combinations of natural compounds, such as organic acids, sugars, alcohols, and amino acids. This study optimized NaDES (sorbitol, citric acid, and glycine) efficiency and compared it to that of 70% methanol solution in extracting total soluble phenolic compounds (TSPCs) from six flours matrices—corn, buckwheat, biofortified orange sweet potato, red lentil, Sudan grass, and chickpea—before and after thermoplastic extrusion cooking. Quantification was performed using the Folin–Ciocalteu method, with statistical analysis at the 10% significance level. In general, the methanolic extracts showed higher TSPC levels in the raw materials, whereas the levels were higher in NaDESs for legumes. After extrusion, a reduction in the TSPC levels was observed, except in the sweet potato. Multivariate analysis (PLS-DA and heatmap) distinguished the raw and extruded samples, revealing structural and chemical changes from thermal processing. The AGREE scores were 0.7 (NaDES) and 0.54 (methanol), favoring NaDES. The BAGI score (75.0) confirmed the method’s robustness and suitability for sustainable analytical applications.

## 1. Introduction

Phenolic compounds are secondary metabolites widely distributed among plants, characterized by a chemical structure comprising an aromatic ring conjugated with one or more hydroxyl groups. Their biosynthesis primarily occurs through the shikimic acid and malonic acid metabolic pathways [1,2].

The structural configuration of these compounds is closely linked to their bioactive properties, particularly their antioxidant capacity, which underscores their importance in food preservation and applications related to human health. Consequently, there is a broad consensus that phenolic compounds contribute to the color and flavor of foods, offering additional health benefits to consumers [3].

Although the health benefits of phenolic compounds have been extensively documented, there has been a concurrent increase in research aimed at their incorporation into food products. However, the solvents traditionally used for the extraction of these compounds are mostly derived from petrochemical sources and are volatile, flammable, toxic to both consumers and operators, and harmful to the environment [4,5,6,7,8]. In this context, the pursuit of solvents that align with the principles of Green Chemistry has garnered increasing attention in both academic research and industrial applications. According to Wang et al. [7] solvents considered compliant with the Green Chemistry principles should be derived from renewable resources, exhibit chemical and thermal stability, permit recovery and reuse, and possess minimal volatility and non-flammability. These attributes are essential to minimizing air pollution and enhancing the overall safety of chemical processes. In this regard, natural deep eutectic solvents (NaDESs) meet these principles and have been highlighted as a promising alternative. These solvents are composed of binary or ternary systems composed of substances such as amino acids, sugars, organic acids, and alcohols, with atoxic properties and Generally Recognized as Safe (GRAS) [9,10]. Moreover, NaDESs offer adjustable viscosity, are biodegradable, easy to prepare, and enable extraction approaches that are compatible with various types of raw material [5].

These solvent systems offer several notable advantages over the traditional solvents, including a reduced environmental impact, high efficiency in the solubilization and stabilization of bioactive compounds, low-level toxicity or atoxic properties, and economic viability. Moreover, NaDESs are widely applied in the extraction of phenolic compounds due to their ability to interact with target biomolecules via hydrogen bonding and to disrupt the cellular matrix, thereby facilitating the release of desired metabolites [11].

The Folin–Ciocalteu method is the most widely used assay for the quantification of total phenolic compounds and is based on a redox reaction. In this reaction, phenolic compounds act as reducing agents capable of converting the metallic ions of molybdenum and tungsten present in Folin–Ciocalteu reagent into lower oxidation states. This reduction results in the formation of a blue complex, whose intensity is enhanced by the addition of a base, typically sodium carbonate [12]. The resulting blue coloration is measured by spectrophotometry, and the total phenolic content is determined using a calibration curve, usually constructed with gallic acid as the standard.

However, the quantification of total phenolic content using this method may vary depending on the characteristics of the raw material, the extraction technique and the solvent employed. In this context, the present study aimed to optimize the extraction of soluble total phenolic compounds (TSPCs) from various matrices, including corn (*Zea mays*), buckwheat (*Fagopyrum esculentum* Moench), biofortified orange sweet potato (*Ipomoea batatas* (L.) Lam), red lentil (*Lens culinaris*), Sudan grass (*Sorghum sudanense* (Piper) Stapf), and chickpea (*Cicer arietinum* L.), using NaDESs.

To achieve this goal, a ternary solvent system composed of sorbitol (6 M), citric acid (60 mM), and glycine (300 mM) was optimized for the extraction of TSPCs, along with the determination of the optimal number of extraction cycles for exhaustive recovery. In addition, the TSPC levels were quantified in both the raw materials and their corresponding extruded products. The performance of the NaDES system was compared to a conventional 70% methanol solution, revealing differences in extraction efficiency depending on the matrix and the processing conditions. Finally, the critical evaluation of the environmental impact of the process was carried out using Green Chemistry metrics, highlighting the sustainability and efficiency of the proposed extraction methodology.

## 2. Materials and Methods

### 2.1. Raw Materials and Reagents

The raw materials used in this study included flours and extrudates of corn, buckwheat, biofortified orange sweet potato, red lentil, Sudan grass, and chickpea. Prior to processing, the samples were milled using a Wiley-type macromill (TE-650, Tecnal, Piracicaba, SP, Brazil) equipped with a 0.3 mm mesh sieve, yielding fine and homogeneous flours. The flours were then stored at –18 °C in high-density polyethylene bags, protected from light. After seven days of storage, the samples were transported to the Laboratory of Cereals, Roots, and Tubers at the University of Campinas, where they were immediately subjected to extrusion processing.

### 2.2. Experimental Design

This study was conducted using the Response Surface Methodology (RSM), applying a constrained Simplex-Centroid Mixture Design (Table 1) to evaluate the different proportions of extraction solutions for the trials following the approach described by Rodrigues and Iemma [13]. The independent variables considered were 3 M sorbitol solution (x_1_), 60 mM citric acid (x_2_), and 300 mM glycine (x_3_).

The use of sorbitol, citric acid, and glycine as constituents of NaDESs is considered a scientifically relevant and sustainable approach for the extraction of phenolic compounds from cereals and legumes. These primary metabolites, such as sorbitol (a polyalcohol), citric acid (an organic acid), and glycine (an amino acid), are combined to form eutectic mixtures with tunable polarity and enhanced hydrogen-bonding capacity, which are essential for the efficient solubilization of phenolic compounds with varying chemical structures. Sorbitol is employed to modulate viscosity and to facilitate interactions with the hydroxyl groups present in phenolic molecules. Citric acid is used to increase the polarity of the solvent system, thereby improving the extraction of hydrophilic phenolic species. Glycine provides zwitterionic properties, which enhance hydrogen-bonding interactions and contribute to the disruption of plant cell wall structures, promoting the release of bound phenolics. Furthermore, these NaDES systems are regarded as environmentally friendly due to their low-level toxicity, biodegradability, and potential food-grade compatibility, making them suitable for applications in the development of functional foods and nutraceuticals. As a result, the combination of sorbitol, citric acid, and glycine in NaDES formulations is recognized as an innovative and efficient strategy for the recovery of phenolic antioxidants from plant matrices, while supporting the principles of green extraction technologies [11].

### 2.3. Extraction of TSPC and Absorbance Quantification

**Figure 1:**
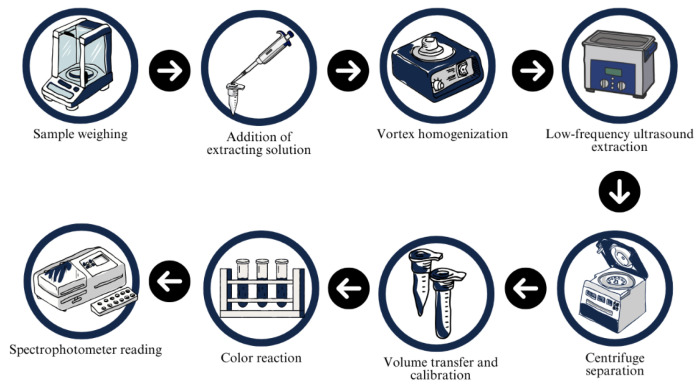
Schematic flowchart of extraction of total phenolic compounds using natural deep eutectic solvents.

The extraction of TSPC from the raw materials was performed using low-frequency ultrasound-assisted extraction, followed by spectrophotometric quantification employing Folin–Ciocalteu reagent. For extraction, 200 mg of each flour was accurately weighed into Eppendorf-type microtubes, followed by the addition of 1200 µL of the NaDES solution prepared according to the Simplex-Centroid Mixture Design (Table 1). The samples were initially homogenized by an NA 3600 vortex mixer (Norte Científica, Araraquara, Brazil) to ensure the complete dispersion of solids in the extraction medium. Subsequently, extraction was carried out using a CBU/100/3LDG low-frequency ultrasound (Planatec, São Paulo, Brazil) operating at 40 kHz and 100 W at room temperature (~20 °C), following a protocol consisting of two cycles of 30 min each, with a 20 min resting interval between cycles. Following extraction, the samples were centrifuged at 5000 rcf for 10 min at 20 °C using an SL-5GR centrifuge (Spinlab, Ribeirão Preto, Brazil). The supernatant phase was recovered, and the final volume was adjusted to 2000 µL with the corresponding extraction solution. For the colorimetric assay, 100 μL of the extract was mixed with 250 μL of 0.2 N Folin–Ciocalteu reagent, 3 mL of distilled water, and 1 mL of 15% (*w*/*v*) sodium carbonate solution. The mixture was then incubated in a dark environment for 30 min to allow for complete color development. Absorbance was measured at 750 nm using a SpectraMax^®^ Paradigm^®^ microplate spectrophotometer (Molecular Devices, San Jose, USA). The literature shows inconsistencies regarding the wavelength employed in the Folin–Ciocalteu method [14,15,16,17]. Therefore, to establish the optimal reading conditions for the present study, an absorbance scan was conducted in the range of 600 to 900 nm using a Cary 60 UV–Vis spectrophotometer (Agilent Technologies, Santa Clara, CA, USA). The wavelength corresponding to the maximum absorbance peak was identified at 750 nm, and subsequently adopted for all measurements. All analyses were performed in triplicate to ensure analytical precision. A detailed schematic representation of the experimental procedure is provided in Figure 1.

### 2.4. Optimization of Extraction Cycles

Following the optimization of the extraction solution, the samples underwent exhaustive ultrasound-assisted extraction cycles to ensure the maximal leaching of TSPCs from the analyzed matrices. This procedure involved a total of eight ultrasound-assisted extraction cycles, aiming to achieve the cumulative extraction of at least 95% of the TSPCs present in the samples. The extraction protocol was designed to optimize the solubilization efficiency of the target compounds, while preventing the saturation of the extraction medium. Each low-frequency ultrasound-assisted extraction cycle was performed for 5 min. The extraction performance was monitored and represented through spectrophotometric profile graphs expressed in absorbance (ABS, at 750 nm) and as cumulative absorbance percentages (%).

### 2.5. Production of Expanded Extrudates

The expanded extrudates were produced as described by Araújo et al. [18]. The flours were conditioned to a moisture content of 20% (*w*/*w*) and processed using a co-rotating twin-screw extruder ZSK-30 (Werner & Pfleiderer, Karlsruhe, Germany) equipped with four heating zones set at 60, 80, 115, and 140 °C at a feed rate of 11 kg·h^−1^, and a screw speed of 250 rpm. The extruder screw had a length of 870 mm and a diameter of 30 mm, resulting in a length-to-diameter (L/D) ratio of 29. A die with two circular openings with 4 mm in diameter each was employed, and the samples were manually cut to be ~200 mm in length. Following extrusion, the products were dried in an oven at 85 °C, cooled to room temperature (~25 °C), manually cut, and packaged in biaxially oriented polypropylene (BOPP) bags. The extrudates were ground using a TE-350 ball mill (Tecnal, Piracicaba, Brazil) to reduce the particle size to below 150 µm and were subsequently stored at room temperature protected from light.

### 2.6. Quantification of TSPC

The TSPCs in the flours and their respective extrudates were quantified using the Folin–Ciocalteu method. Extracts were obtained using the optimized NaDES solutions and each respective cycle and for 70% (*v*/*v*) methanol, which served as the reference solvent. A colorimetric assay was performed as described for TSPC absorbance quantification.

Calibration curves were prepared using gallic acid standards, comprising eight points ranging from 0 to 600 mg·L^−1^ for corn (y = 0.0006x + 0.0347; r = 0.9814), buckwheat (y = 0.0006x + 0.0399; r = 0.9853), biofortified orange sweet potato (y = 0.0006x + 0.024; r = 0.9882), red lentil (y = 0.0007x + 0.0205; r = 0.9834), Sudan grass (y = 0.0005x + 0.0186; r = 0.983), and chickpea (y = 0.0007x + 0.0234; r = 0.9835). Additionally, a calibration curve for extracts obtained with 70% (*v*/*v*) methanol was constructed (y = 0.0007x + 0.0149; r = 0.9978). All analyses were performed in triplicate, with the results expressed as mg gallic acid equivalent (GAE) per 100 g in dry weight.

### 2.7. Determination of the AGREE Index (Analytical Greenness Metric Approach)

The environmental impact associated with the extraction and quantification of TSPC using NaDESs and 70% methanol was evaluated using the AGREE index, in accordance with the methodology proposed by Pena-Pereira et al. [19]. The AGREE index is based on the 12 principles of Green Analytical Chemistry, which are weighted according to their relevance to the specific methodology and translated into a unified scale from 0 to 1. The final score was calculated by considering the relative importance of the principles applicable to the context under evaluation.

### 2.8. Determination of the BAGI (Blue Applicability Grade Index)

The applicability of the analytical method used for the extraction and quantification of total soluble phenolic compounds was evaluated using the BAGI (Blue Applicability Grade Index). According to the methodology proposed by Manousi et al. [20], the BAGI is based on ten distinct criteria that encompass the structure, accessibility, and operational robustness of a method. The final score is expressed on a unified scale from 0 to 100, allowing for the clear and quantitative assessment of the suitability, reliability, and feasibility of the method used.

### 2.9. Statistical Analysis

The data from the Simplex-Centroid Mixture Design were analyzed using the Response Surface Methodology (RSM) following Equation (1) as described by Rodrigues and Iemma [13] implemented using Statistica StatSoft, Inc. (Tulsa, OK, USA) (2004). Regression coefficients and analysis of variance (ANOVA) were assessed at the 10% significance level. Extraction solution optimization was carried out using Design Expert 6.0 software based on the methodology of Derringer and Suich [21]. The quantification data of total soluble phenolics from the raw materials and the extrudates obtained with optimized NaDESs and 70% methanol solution were compared using Student’s t-test (*p* < 0.05). For the multivariate analysis, MetaboAnalyst software (version 6.0) was employed (*p* < 0.05). The experimental data were subjected to Partial Least Squares Discriminant Analysis (PLS-DA) and heatmap generation to evaluate the variability in TSPCs between the extractions performed with NaDESs and 70% methanol following the methodology described by Massaretto et al. [22].

*Y* = *β*_1_x_1_ + *β*_2_x_2_ + *β*_3_x_3_ + *β*_12_x_1_x_2_ + *β*_13_x_1_x_3_ + *β*_23_x_2_x_3_ + *β*_123_x_1_x_2_x_3_ + ε (1)

where *Y* is the dependent variable; *β*_i_ and *β*_ij_ represent coefficients for pseudo-components and their binary and ternary interactions, respectively; *i* and *j* denote the independent variables in coded form; and *ε* is experimental error.

## 3. Results and Discussion

### 3.1. Optimization of the Extracting Solution

**Figure 2:**
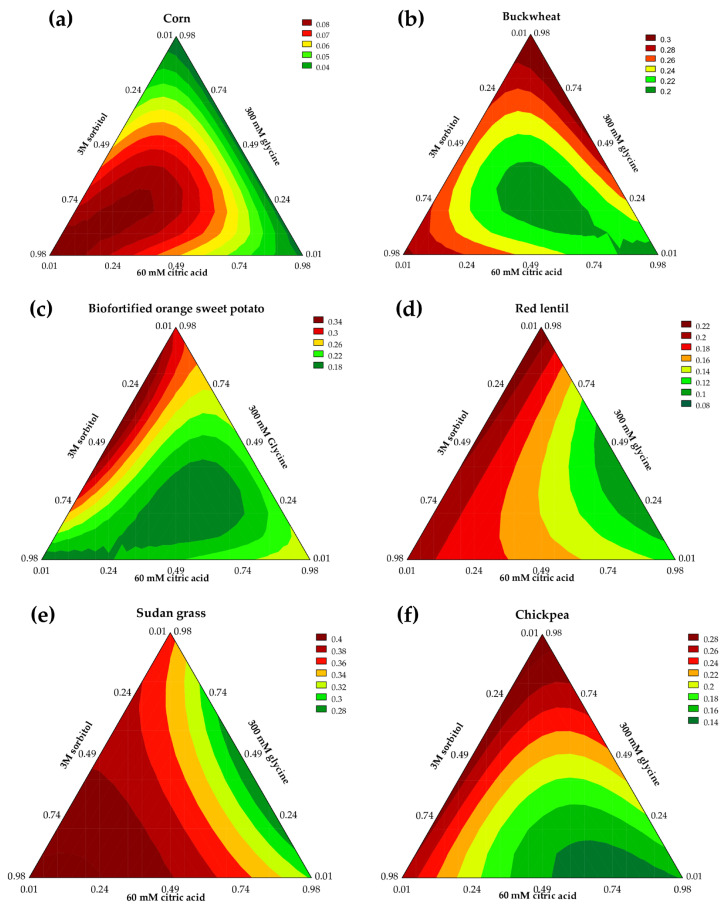
Contour plots of absorbance responses for total phenolic compounds extracted with natural deep eutectic solvents from corn (**a**), buckwheat (**b**), biofortified orange sweet potato (**c**), red lentil (**d**), Sudan grass (**e**), and chickpea (**f**). Regression models and ANOVA were evaluated at 10% significance level (*p* ≤ 0.10).

The absorbance values of the TSPC extracts from corn ranged from 0.026 to 0.092 (at 100 mg·mL^−1^). The predictive mathematical model explained 86.17% of the variance, with an F_calc_/F_tab(3;8;0.10)_ ratio of 6.65 and *p* < 0.001, indicating a highly significant fit. According to the contour plot (Figure 2a) and the mathematical model for corn (Equation (2)), the ternary mixture of 3 M sorbitol, 60 mM citric acid, and 300 mM glycine solutions demonstrated the best performance for solubilizing TSPCs (*β*_123_ = 0.67, *p* < 0.001), with sorbitol showing the strongest individual contribution to extraction efficiency (*β*_1_ = 0.08, *p* < 0.001). The superior extraction capacity of sorbitol is likely due to its chemical interactions with phenolic compounds through hydrogen bonding. Being a highly hydrophilic polyol, sorbitol possesses six hydroxyl groups (-OH), which enhance these interactions and facilitate phenolic compound solubilization, thereby promoting efficient extraction [23].

*Y* = 0.08x_1_ + 0.03x_2_ + 0.03x_3_ + 0.67x_1_x_2_x_3_ (2)

where *Y* denotes the absorbance of the extract corresponding to the total phenolic compounds from corn based on the initial concentration of 100 mg of dry matter per mL. The variables x_1_, x_2_, and x_3_ represent the coded levels of the independent factors, sorbitol (3 M), citric acid (60 mM), and glycine (300 mM), respectively.

The extracts obtained from buckwheat exhibited absorbance values ranging from 0.177 to 0.293 (100 mg·mL^−1^), which were directly influenced by the solvents employed, as well as by the binary and ternary mixtures. The mathematical model accounted for 91.88% of the variability in the data and was deemed predictive according to ANOVA, presenting an F_calc_/F_tab(5;7;0.10)_ of 5.50 with *p* < 0.001. As indicated by the contour plot (Figure 2b) and supported by the mathematical model (Equation (3)), the most effective extraction of phenolic compounds from buckwheat occurred with the highest proportion of glycine at 300 mM (*β*_3_ = 0.30, *p* < 0.001), followed by sorbitol at 3 M (*β*_1_ = 0.28, *p* < 0.001) and citric acid at 60 mM (*β*_2_ = 0.18, *p* < 0.001). Pires et al. [24] investigated the extraction of phenolic compounds from pitaya using an NaDES composed of lactic acid and glycine. Fourier-transform infrared spectroscopy confirmed the formation of hydrogen bonds between the hydroxyl group of lactic acid and the amino and carboxyl groups of glycine, promoting a stable and efficient solvent structure. This interaction enhanced the extraction of flavonoids, phenolic acids, and other bioactive metabolites. Compared to the other NaDES formulations, the glycine-based system demonstrated superior extraction efficiency, underscoring its role as a co-solvent capable of modulating polarity and improving the solubilization of phenolic compounds.

*Y* = 0.28x_1_ + 0.18x_2_ + 0.30x_3_ − 0.15x_1_x_3_ + 0.15x_2_x_3_ − 1.76x_1_x_2_x_3_ (3)

where *Y* denotes the absorbance of the extract corresponding to the total phenolic compounds from buckwheat based on an initial concentration of 100 mg of dry matter per mL. The variables x_1_, x_2_, and x_3_ represent the coded levels of the independent factors, sorbitol (3 M), citric acid (60 mM), and glycine (300 mM), respectively.

The absorbance of TSPCs in the extracts from biofortified orange sweet potato ranged from 0.075 to 0.169 nm (100 mg·mL^−1^). The mathematical model (Equation (4)) describing extraction accounted for 96.00% of the data variability and was deemed predictive. ANOVA revealed an F-ratio = 10.73, with a *p*-value < 0.001, indicating high statistical significance and supporting the generation of the contour plot (Figure 2c). Among the components evaluated, the binary interaction between sorbitol and glycine (*β*_12_ = 0.45, *p* < 0.001) resulted in the highest extraction yield of TSPCs. In contrast, the ternary interaction among all the components (*β*_123_ = −2.33, *p* < 0.001) reduced the extraction efficiency, suggesting that this combination was not favorable. Pires et al. [24] reported that although NaDESs exhibit promising extraction potential, their combinations and efficiency may be inferior to those of conventional solvents. This observation implies that despite the advantages of NaDESs regarding sustainability and selectivity, their extractive capacity may not surpass that of the traditional methods, potentially limiting their applicability in scenarios requiring higher yields. Conversely, the use of NaDESs may be particularly advantageous when the aim is to employ the extracts in food, cosmetic, or pharmaceutical formulations.

*Y* = 0.16x_1_ + 0.24x_2_ + 0.30x_3_ + 0.45x_1_x_3_ − 0.19x_2_x_3_ − 2.33x_1_x_2_x_3_ (4)

where *Y* denotes the absorbance of the extract corresponding to the total phenolic compounds from biofortified orange sweet potato based on an initial concentration of 100 mg of dry matter per mL. The variables x_1_, x_2_, and x_3_ represent the coded levels of the independent variables sorbitol (3 M), citric acid (60 mM), and glycine (300 mM), respectively.

The absorbance of TSPCs in the red lentil extracts ranged from 0.089 to 0.225 (100 mg·mL^−1^). The predictive mathematical model (Equation (5)) accounted for 91.34% of data variability, with ANOVA revealing an F_calc_ value 11.26 times greater than F_tab(3;9;0.10)_ and a significance level below 0.001. As depicted in the contour plot (Figure 2d), the highest extraction yield of TSPCs was associated with the greatest proportion of glycine (*β*_3_ = 0.22, *p* < 0.001) in the extraction solvent, followed by sorbitol (*β*_1_ = 0.18, *p* < 0.001) and citric acid (*β*_2_ = 0.11, *p* < 0.001).

*Y* = 0.18x_1_ + 0.11x_2_ + 0.22x_3_ − 0.32x_2_x_3_ (5)

where *Y* denotes the absorbance of the extract corresponding to the total phenolic compounds from red lentil based on an initial concentration of 100 mg of dry matter per mL. The variables x_1_, x_2_, and x_3_ represent the coded levels of the independent factors sorbitol (3 M), citric acid (60 mM), and glycine (300 mM), respectively.

The absorbance of TSPCs in the Sudan grass extracts ranged from 0.270 to 0.407 (100 mg·mL^−1^), being directly influenced by the individual solvents as well as their binary and ternary mixtures. A total of 95.49% of the extraction results was explained through the predictive mathematical model (Equation (6)), with ANOVA yielding an F_calc_/F_tab(5;7;0.10)_ ratio of 10.29 (*p* < 0.001), confirming the model’s predictive validity. As illustrated by the contour plot (Figure 2e) and supported by the mathematical model, the highest level of extraction of TSPCs was attained with the ternary mixture of the evaluated components (*β*_123_ = 0.53, *p* = 0.067). Among the individual effects, sorbitol contributed most significantly (*β*_1_ = 0.40, *p* < 0.001), followed by glycine (*β*_3_ = 0.35, *p* < 0.001) and citric acid (*β*_2_ = 0.29, *p* < 0.001), enhancing the solubilization of TSPCs.

*Y* = 0.40x_1_ + 0.29x_2_ + 0.35x_3_ + 0.12x_1_x_2_ − 0.24x_2_x_3_ + 0.53x_1_x_2_x_3_ (6)

where *Y* represents the absorbance of the extract corresponding to the total phenolic compounds in Sudan grass based on an initial concentration of 100 mg dry sample per mL. The variables x_1_, x_2_, and x_3_ denote the coded levels of the independent factors sorbitol (3 M), citric acid (60 mM), and glycine (300 mM), respectively.

The absorbance of the chickpea extracts varied from 0.147 to 0.315 nm (100 mg·mL^−1^). The mathematical model (Equation (7)) accounted for 86.58% of the data variability and was deemed predictive, as confirmed by the ANOVA results showing an F_calc_/F_tab(4;8;0.10)_ ratio of 4.59 with *p* = 0.001. As depicted in the contour plot (Figure 2f), glycine (*β*_3_ = 0.29, *p* < 0.001) exerted the strongest positive effect on the extraction of TSPC, followed by sorbitol (*β*_1_ = 0.26, *p* < 0.0001) and citric acid (*β*_2_ = 0.14, *p* < 0.001). Despite the effective individual contributions of these pseudocomponents toward solubilizing phenolic compounds, their binary combinations were less effective, exemplified by the interaction between sorbitol (3 M) and citric acid (60 mM) (*β*_12_ = −0.22, *p* = 0.098).

Y = 0.26x_1_ + 0.14x_2_ + 0.29x_3_ − 0.22x_1_x_2_ (7)

where *Y* represents the absorbance of the extract corresponding to the total phenolic compounds in chickpea based on an initial concentration of 100 mg of dry sample per mL; x_1_, x_2_, and x_3_ denote the coded levels of the independent variables, sorbitol (3 M), citric acid (60 mM), and glycine (300 mM), respectively.

The effectiveness of the Folin–Ciocalteu method in quantifying total phenolic compounds is well-established. NaDESs characterized by their diverse compositions may demonstrate selective extraction capabilities [25]. Consequently, the resulting extracts may vary not only in total phenolic content, but also in their specific phenolic profiles. However, a detailed qualitative characterization aimed at identifying the individual phenolic compounds was not performed in the present study. This omission represents a significant limitation that should be addressed in future research to improve the understanding of the chemical composition of NaDES extracts and to enhance the applicability of the developed extraction methods.

### 3.2. Numerical Optimization and Validation of the Mathematical Models

The optimization goals were established within the study range for the coded levels (from 0 to 1) of the independent variables, each assigned an importance level of three. For absorbance, optimization aimed to maximize the absorbance at 750 nm (100 mg·mL^−1^), with an importance level of five. The importance values range from one (lowest importance level) to five (highest importance level). Based on the results presented in Table 2, the optimized condition for extracting the TSPCs from corn was achieved using a solution composed of 97.82% sorbitol (3 M), 1.18% citric acid (60 mM), and 1.00% glycine (300 mM), resulting in a desirability index of 93.03%.

The efficiency of the optimized extraction system may be attributed to the specific nature of the phenolic compounds present in cereals, which differ from those typically found in fruits and vegetables. In grains, phenolics occur in various forms: soluble-free, soluble-ester, and predominantly insoluble [26]. The insoluble forms are primarily located in the outer layers of the grains, bound to cell walls through covalent linkages with hemicellulose and cellulose, making their release more challenging. Conversely, the soluble forms are more accessible, being associated with internal structures such as proteins, starch, and vacuoles [27].

For buckwheat, the optimal extraction conditions were identified as a mixture containing 98.00% sorbitol (3 M), 1.00% citric acid (60 mM), and 1.00% glycine (300 mM), resulting in a desirability score of 92.72%. This finding is consistent with the predictions from the mathematical model and the contour plots, confirming the efficiency of the extraction system in maximizing the yield of TSPCs from this matrix. The high extraction efficiency can be attributed both to the composition of the solvent mixture and to the intrinsic phytochemical profile of buckwheat, which is known for its rich content of flavonoids and phenolic acids [28]. The major phenolic compounds include rutin, epicatechin, catechin, vitexin, quercetin, gallic acid, and protocatechuic acid. Among these, rutin, epicatechin, and catechin are often highlighted as the main contributors to antioxidant activity, owing to their potent free radical scavenging abilities and structural stability [29].

For red lentils, the optimal extraction condition was determined using a formulation containing 1.02% sorbitol (3 M), 1.00% citric acid (60 mM), and 97.98% glycine (300 mM), achieving a desirability score of 96.11%. The high extraction efficiency is likely due to glycine’s strong affinity as a solubilizing agent in this matrix, which facilitates the release of phenolic compounds. This efficiency is particularly significant given the composition of lentils, which beyond their nutritional value characterized by high levels of protein, carbohydrates, and lipids [30], are also known as a rich source of bioactive compounds. Among these, flavonoids, phenolic acids, lignans, and stilbenes are prominent compounds noted for their antioxidant and anti-inflammatory properties, as well as their potential role in preventing chronic diseases [31]. However, the bioavailability of these compounds may be limited by antinutritional factors, such as tannins, phytic acid, protease inhibitors, and lectins, which can bind to polyphenols and impede their release [23].

The optimized extraction solution for chickpeas comprised 1.00% sorbitol (3 M), 1.00% citric acid (60 mM), and 98.00% glycine (300 mM), achieving a desirability score of 85.98%. This outcome suggests that glycine played a pivotal role in extracting the TSPCs from this matrix, likely enhancing both the dissolution and stabilization of the phenolic compounds. Chickpeas are a significant source of phytochemicals with notable antioxidant properties, particularly flavonoids [32]. This legume contains phenolic compounds across several classes, including hydroxybenzoic acids, hydroxycinnamic acids, isoflavonoids, and flavonoids, all of which contribute to its health-promoting effects [33]. However, the effective release of these compounds can be challenged by the complexity of the matrix and the variability among cultivars, underscoring the need to develop extraction solutions specifically tailored to the unique composition of each grain.

The extracted TSPCs from biofortified orange sweet potato, as detailed in Table 2, were distinct compared to those of the other matrices evaluated. The optimized solvent composition consisted of 1.00% sorbitol (3 M), 98.00% citric acid (60 mM), and 1.00% glycine (300 mM), yielding a desirability score of only 45.67%. This relatively low value suggests that the solvent system may have been insufficiently effective in solubilizing the phenolic compounds in the orange sweet potato, potentially due to interactions with the starchy matrix and the complexation of phenolic compounds with other biomolecules present in the plant tissue [34]. Despite the limited extraction efficiency observed, orange sweet potato contains a large amount of bioactive compounds, such as phenolic acids and β-carotene (a vitamin A precursor), both linked to various health benefits, including antioxidant, anti-inflammatory, and hepatoprotective effects [35].

For Sudan grass (Table 2), the extracted TSPCs were distinct, with the optimal composition determined as 92.41% sorbitol (3 M), 6.54% citric acid (60 mM), and 1.00% glycine (300 mM), achieving a desirability index of 98.27%. This high desirability value reinforces the effectiveness of the solvent composition in extracting bioactive compounds from this matrix, possibly due to a synergistic interaction between sorbitol and citric acid in releasing the TSPCs.

This outstanding performance can likely be attributed to the distinctive phytochemical profile of grasses in the Poaceae family, to which Sudan grass belongs. Although it has been less extensively studied than grains such as sorghum and Sudan grass shares significant structural and biochemical similarities, it is potentially rich in phenolic compounds, such as ferulic, *p*-coumaric, and caffeic acids, along with flavonoids like luteolin and apigenin. These bioactive compounds are well-known for their potent antioxidant, anti-inflammatory, and cardioprotective effects, which may explain the high extraction efficiency observed. This underscores the promising functional potential of this forage grass in the development of functional foods and nutraceutical products [36,37]

### 3.3. Optimization of Extraction Cycles

Based on the optimized extraction conditions for each raw material, the samples underwent successive cycles of ultrasound-assisted extraction to ensure the maximal recovery of TSPCs. Exhaustive extraction was defined as the recovery of at least 95% of the TSPCs present in each matrix, with the results expressed as individual absorbance readings for each cycle and cumulative absorbance (%).

**Figure 3:**
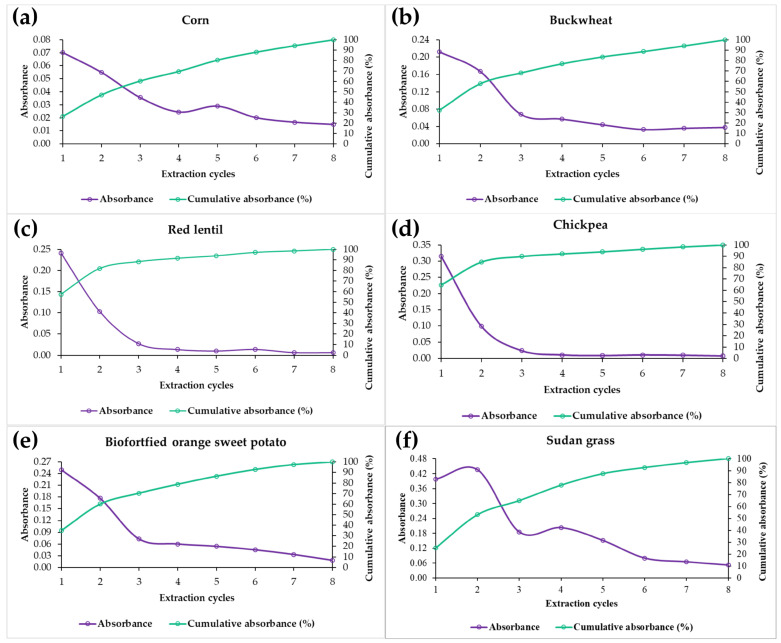
Spectrophotometric absorbance profile at 750 nm of total phenolic compounds from corn (**a**), buckwheat (**b**), red lentil (**c**), chickpea (**d**), biofortified orange sweet potato (**e**), and Sudan grass (**f**) across successive extraction cycles.

Corn exhibited greater resistance to the extraction process, requiring eight cycles to achieve target recovery, as illustrated in Figure 3. The presence of starch within the matrix may have contributed to this resistance by hindering the release of phenolic compounds into the extraction solution. Similarly, buckwheat also required eight cycles, suggesting a potential complexation of phenolic compounds with the macromolecular structures, such as polysaccharides and proteins, which can retard their solubilization [38].

Red lentil and chickpea demonstrated a more efficient extraction profile, reaching 95% recovery of the TSPCs after six cycles. This pattern suggests that the phenolic compounds in these matrices exhibit greater solubility in the optimized extraction solvent or are less tightly bound to structural components that impede their release.

Sudan grass and biofortified orange sweet potato each required seven extraction cycles to achieve the satisfactory recovery of TSPCs, underscoring the significant impact of the food matrix on phenolic compound release. In Sudan grass, the elevated number of cycles may be attributed to the presence of structural fibers, as free phenolic compounds form hydrogen bonds and van der Waals interactions with cell wall components, primarily polysaccharides, such as arabinoxylans, *β*-glucans, and cellulose [39].

For sweet potato, the starch composition likely contributed to phenolic retention, given that the supramolecular structure of starch profoundly affects these interactions. Phenolic entrapment can occur due to the granular organization of starch within the matrix, which influences compound accessibility and extraction efficiency, particularly in its native or retrograded state, where the polymer network can encapsulate bioactive compounds and diminish their solubility [39].

### 3.4. Quantification of TSPCs

**Figure 4:**
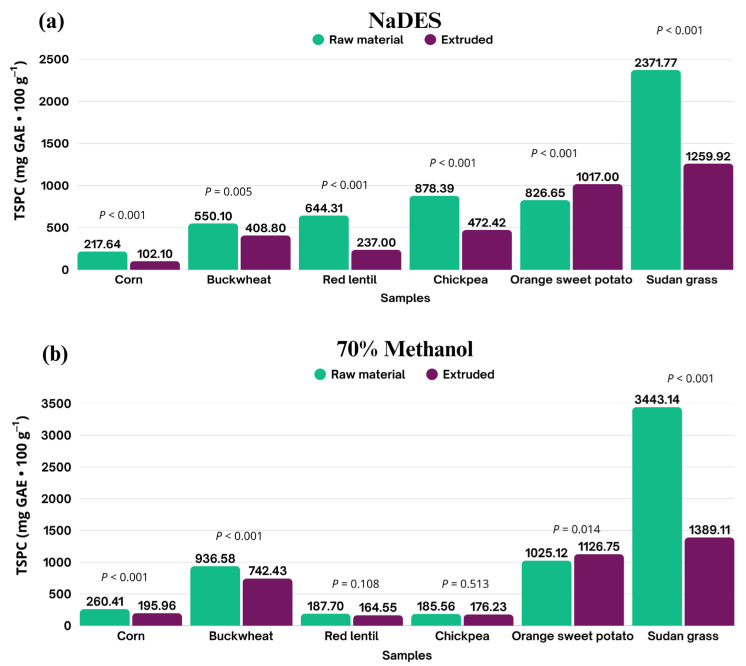
Total phenolic compound contents extracted by natural deep eutectic solvents (**a**) and 70% methanol (**b**) from raw materials and their respective extrudates. Statistical differences were determined by *t*-test (*p* ≤ 0.05).

The extraction of phenolic compounds using NaDESs and 70% methanol revealed significant variations among the analyzed samples, reflecting differences in the chemical composition of the materials, as well as the extraction efficiency of each solvent. Quantification is expressed in milligrams per 100 g of dry basis (mg GAE·100 g^−1^; d.b.), allowing for accurate comparisons between the samples before and after extrusion (Figure 4).

The TSPC content in corn extracted with the NaDESs showed a marked decrease from 217.64 ± 28.55 to 102.10 ± 14.34 mg GAE·100 g^−1^ following extrusion, corresponding to a loss of 53.09%. A similar trend was observed in the methanol extracts (Figure 4b), with the levels declining from 260.41 ± 10.25 to 195.96 ± 17.79 mg GAE·100 g^−1^, representing a 24.75% reduction (*p* < 0.001). Despite these losses in both the solvent systems, the methanolic extracts still demonstrated a higher yield, indicating greater efficiency in extracting total phenolics from the raw material. The reduction in TSPC total due to processing is likely attributable to the high susceptibility of these compounds to thermal degradation and the intense mechanical forces involved during extrusion, such as shear stress and the structural disruption of the matrix, as reported by Šárka et al. [40].

In buckwheat, the losses were notably lower; the TSPC total extracted with NaDESs decreased from 550.10 ± 68.67 to 408.80 ± 76.08 mg GAE·100 g^−1^ (−25.68%), while the amount of methanolic extracts declined from 936.58 ± 36.31 to 742.43 ± 34.97 mg GAE·100 g^−1^ (−20.73%). Despite the smaller reduction, the methanolic extracts consistently exhibited higher phenolic contents than those obtained with NaDESs both before and after extrusion, indicating the stronger affinity of the hydroalcoholic solvent for the phenolics in this matrix. The comparatively lower loss suggests the greater stability of phenolic compounds during processing, likely due to the buckwheat matrix structure providing enhanced protection against thermal degradation. This stability is important because phenolics in cereals and pseudocereals often exist in conjugated polymeric forms, which limit their bioavailability and bioaccessibility. Processing techniques such as thermoplastic extrusion, alongside germination, fermentation, and hydrolysis have been explored to release these compounds and improve their bioavailability, minimizing the losses of thermosensitive phenolics, while boosting their antioxidant and health-promoting effects [41,42].

For biofortified orange sweet potato, an increase in the TSPC levels was observed post-extrusion from 826.65 ± 46.28 to 1017 ± 80.33 mg GAE·100 g^−1^ (d.b.) with the NaDESs (+23.03%) and from 1025.12 ± 52.10 to 1126.75 ± 49.69 mg GAE·100 g^−1^ with methanol (+9.91%). This increase is likely attributable to the disruption of the starch matrix during extrusion, which facilitates the release of phenolic compounds previously bound in complexes with starch or cell wall components, thereby enhancing their accessibility for extraction and potentially improving their bioavailability. Additionally, the retention of thermoresistant phenolic compounds may have been promoted by biofortification [40].

Conversely, as a starchy raw material highly susceptible to the Maillard reaction during extrusion, the observed increase in phenolic compound content in sweet potato may also be influenced by the formation of Maillard reaction products [43]. Furthermore, the high content of reducing sugars may have contributed to an overestimation of the total phenolic content, as these sugars are capable of reducing Folin–Ciocalteu reagent, leading to increased absorbance and an apparent elevation in phenolic levels [44,45].

During thermal processing, the Maillard reaction, a complex series of non-enzymatic browning reactions, occurs between the reducing sugars (such as glucose and fructose) and the free amino groups of amino acids and proteins. The initial step involves the formation of Amadori rearrangement products, which subsequently degrade to form reactive intermediates like furfurals and quinones [46]. These intermediates possess strong reducing properties and play a key role in the synthesis of melanoidins, high-molecular-weight polymers responsible for the characteristic brown coloration and antioxidant activity observed in thermally processed foods [47].

Although the Folin–Ciocalteu assay is widely used for estimating the total phenolic content (TPC), it is not specific to phenolic compounds. It also responds to other reducing substances, including reducing sugars and Maillard-derived compounds. These non-phenolic compounds not only exhibit antioxidant activity, but also absorb strongly at the assay’s detection wavelength (~750 nm), potentially leading to the overestimation of phenolic content [48]. Therefore, the increase in TPC observed in sweet potato after extrusion may be partially attributed to these interfering compounds. To obtain a more accurate assessment of the phenolic profile and to distinguish true phenolics from Maillard-related interferences, future studies should employ complementary analytical techniques such as High-Performance Liquid Chromatography.

For red lentil, the concentration of phenolic compounds extracted using NaDESs was 644.31 ± 77.34 mg GAE·100 g^−1^ (dry basis) in the non-extruded sample, decreasing to 237.28 ± 84.05 mg GAE·100 g^−1^ (dry basis) following the extrusion process, corresponding to a 63.17% reduction. Nevertheless, NaDES extraction proved to be more efficient than methanolic extraction, which yielded lower values of 187.70 ± 20.18 mg GAE·100 g^−1^ (dry basis) in the non-extruded sample and 164.55 ± 23.20 mg GAE·100 g^−1^ (dry basis) in the extruded product.

A similar pattern was observed for chickpea, where NaDES extraction resulted in a phenolic concentration of 878.39 ± 78.12 mg GAE·100 g^−1^ (dry basis) in the non-extruded sample and 472.42 ± 66.94 mg GAE·100 g^−1^ (dry basis) after extrusion, representing a 46.22% reduction. In contrast, methanolic extraction yielded considerably lower concentrations, with 185.56 ± 15.95 mg·100 g^−1^ (dry basis) prior to and 176.23 ± 15.90 mg GAE·100 g^−1^ (dry basis) following thermal processing. As with red lentil, the use of NaDESs demonstrated superior extraction efficiency for phenolic compounds under both the conditions.

The reduction in phenolic content observed in both the legumes can be attributed, at least in part, to the thermal effects of extrusion, which promote the degradation of these compounds or facilitate their interaction with proteins and polysaccharides, leading to the formation of complexes that are less accessible for extraction [40,49,50,51]. Nevertheless, the results demonstrate that NaDES-type solvents are more effective at disrupting these phenolic–matrix interactions, thereby improving the solubilization and recovery of the compounds even after processing. This superior performance highlights the critical role of solvent selection, particularly when dealing with protein-rich matrices, such as chickpea and red lentil.

In the case of Sudan grass, the initial phenolic compound content was 2317.77 ± 179.60 mg GAE·100 g^−1^ (dry basis) when extracted using an NaDES-type solvent, which decreased to 1259.90 ± 164.63 mg GAE·100 g^−1^ (dry basis) after extrusion, representing a 45.64% reduction. Conversely, extraction with the methanolic solution yielded an initial concentration of 3443.14 ± 88.29 mg GAE·100 g^−1^ (dry basis), which dropped to 1389.11 ± 68.22 mg GAE·100 g^−1^ (dry basis) post-extrusion, corresponding to a 59.66% loss. Comparing the two extraction systems reveals that although methanol facilitated phenolic recovery in the raw sample, both approaches experienced substantial reductions following extrusion. This decline can be attributed to the partial thermal degradation of phenolic compounds, as well as their potential interaction with the modified plant matrix during processing, ultimately hindering subsequent extraction.

### 3.5. Multivariate Analysis

**Figure 5:**
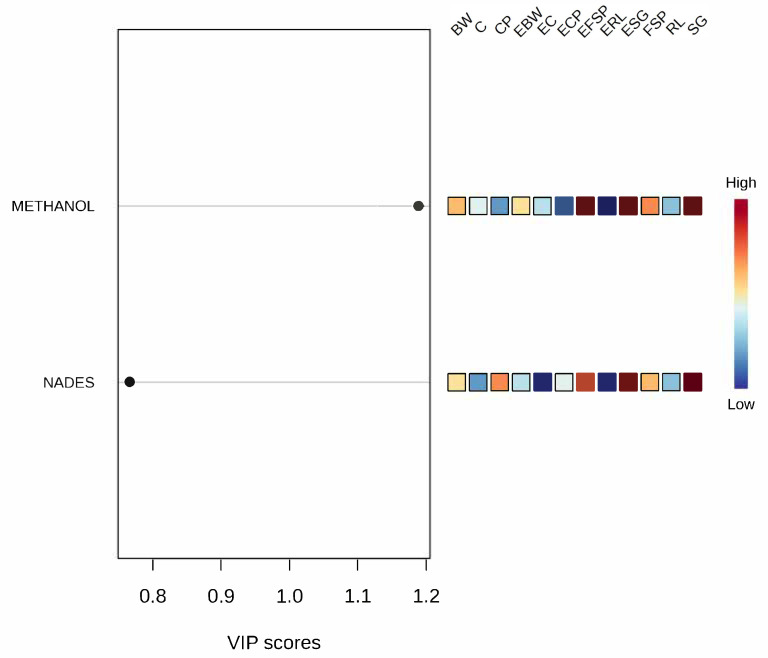
Multivariate analysis by Partial Least Squares Discriminant Analysis (PLS-DA), performed using MetaboAnalyst version 6.0 (*p* ≤ 0.05). Here, BW = buckwheat; C = corn; CP = chickpea; EBW = extruded buckwheat; EC = extruded corn; ECP = extruded chickpea; EFSP = extruded orange biofortified sweet potato; ERL = extruded red lentil; ESG = extruded Sudan grass; FSP = orange-fleshed biofortified sweet potato; RL = red lentil; SG = Sudan grass; VIP = Variable Importance in Projection.

Multivariate analysis using Partial Least Squares Discriminant Analysis (PLS-DA) applied to the phenolic compound extraction data effectively distinguished the samples based on both the nature of the raw material and the impact of extrusion processing. As illustrated in Figure 5, there was a clear separation between the groups of non-extruded samples (buckwheat, corn, chickpea, orange-fleshed biofortified sweet potato, red lentil and Sudan grass) and the respective extruded samples (extruded buckwheat, extruded corn, extruded chickpea, extruded orange biofortified sweet potato, extruded red lentil, extruded Sudan grass), demonstrating that thermal processing significantly affected phenolic compound extraction, irrespective of the solvent employed.

The raw materials were distinctly separated from the extruded samples, indicating that the extractable phenolic composition was significantly altered by extrusion, likely due to thermal degradation, complexation with the plant matrix, or the formation of new derivatives with different chemical properties [40]. Within the extruded group, clear clusters emerged reflecting the similarities between the matrices. The EBW (extruded buckwheat) and EC (extruded corn) samples clustered closely in the discriminant space, suggesting comparable phenolic extraction profiles, likely linked to their predominantly carbohydrate-rich profiles. Likewise, ECP (extruded chickpea) and ERL (extruded red lentil) were grouped, probably due to their high protein content and bioactive compound profiles typical of legumes, resulting in similar solvent interactions after thermal processing [52,53].

The extruded biofortified sweet potato (EFSP) displayed a unique profile compared to those of the other samples, likely due to its distinct composition, particularly the high concentration of carotenoids resulting from biofortification. Among these carotenoids, β-carotene is predominant, often comprising the majority of total carotenoids in orange-fleshed sweet potatoes after maturation. Additionally, smaller amounts of lycopene and *β*-cryptoxanthin are present, while *α*-carotene and lutein are typically absent in these cultivars. The abundant presence of *β*-carotene and other biofortification compounds may affect the efficiency of phenolic extraction, either through chemical interactions or competition for solvents during the extraction processes [54,55].

The extruded Sudan grass (ESG) exhibited a distinct profile relative to those of the other extruded samples, which can be attributed to its unique composition, marked by a high content of structural fibers and a rich concentration of phenolic compounds, characteristics inherent to this grass species. The substantial fiber content, especially lignocellulosic fibers, may impede the release and extraction of certain bioactive compounds, while the phenolic-rich plant matrix further contributes to its distinctive extraction profile. Collectively, these factors account for the distinctive profile of ESG observed in the analyses [37,56].

**Figure 6:**
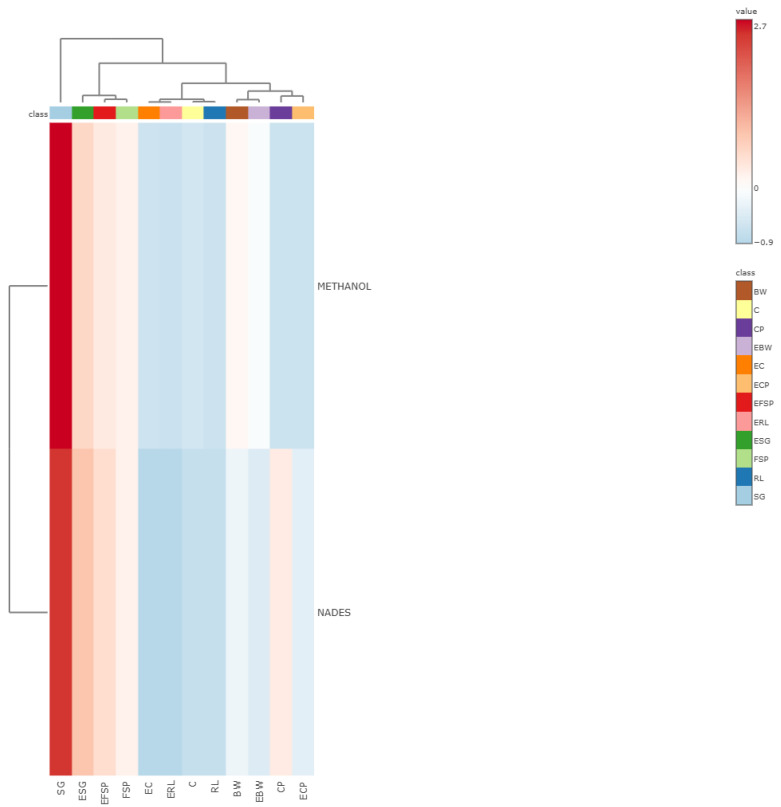
Heatmap illustrating the quantification of phenolic compounds extracted using methanol and natural deep eutectic solvents generated using MetaboAnalyst version 6.0 (*p* ≤ 0.05). Here, BW = buckwheat; C = corn; CP = chickpea; EBW = extruded buckwheat; EC = extruded corn; ECP = extruded chickpea; EFSP = extruded orange biofortified sweet potato; ERL = extruded red lentil; ESG = extruded Sudan grass; FSP = orange biofortified sweet potato; RL = red lentil; SG = Sudan grass.

The heatmap generated from the phenolic compound extraction data using methanolic solvent and NaDESs (Figure 6) reveals a clear differentiation between the profiles extracted by each solvent, as evidenced by the distinct horizontal segmentation between the two extraction conditions. This separation underscores the influence of solvent type on extraction efficiency, with methanol generally proving more effective for most samples, as indicated by the higher intensity values (reddish tones) in the upper region of the heatmap.

Notably, Sudan grass (SG) and its extruded form (ESG) exhibited the highest extraction yields with the methanolic solvent, reflecting a substantial concentration of extractable phenolic compounds within this matrix. This finding aligns with their isolated position observed in the earlier PLS-DA analysis. Such results suggest that this matrix possesses a unique phenolic profile with a strong responsiveness to methanol extraction, likely due to the presence of more polar and free phenolic compounds. Similar observations were reported by Carvalho et al. [37], who documented a greater total phenolic content using reducing agents (2136 mg GAE·100 g^−1^), followed by alkaline agents (1883 mg GAE·100 g^−1^), combined agents (1941 mg GAE·100 g^−1^), and aqueous extraction (1838 mg GAE·100 g^−1^). These results reinforce the notion that phenolic compounds with higher polarity or reduced molecular binding are more efficiently extracted by solvents with strong polar affinity such as methanol [37].

The clustering of EBW, BW, CP, and ECP in NaDES extraction highlights a shared extraction profile among these samples, characterized by relatively low yields for the phenolic compounds. This supports the hypothesis that matrices rich in carbohydrates (e.g., buckwheat) or proteins (e.g., legumes) may experience greater phenolic complexation or reduced accessibility when exposed to eutectic solvents, especially after thermal processing. Furthermore, the biofortified sweet potato (FSP) and its extruded counterpart (EFSP) demonstrated moderate extraction efficiencies with both solvents, with a slightly better performance using methanol. This intermediate extraction profile corroborates their distinct positioning in the multivariate analysis, likely influenced by their high carotenoid and starch contents, which can modulate the release and solubility of phenolic compounds across different solvent systems [54].

Overall, the heatmap reinforces the PLS-DA analysis results by demonstrating that both the matrix composition and extrusion processing significantly impact the efficiency of phenolic compound extraction. Additionally, it underscores solvent selectivity, with methanol achieving higher extraction yields for the samples rich in free and polar phenolics, whereas NaDESs tend to be less effective under these conditions, particularly in starch-rich extrudates. These insights are valuable for optimizing extraction strategies in both analytical and industrial applications aimed at recovering bioactive compounds.

Although the NaDESs exhibited lower extraction yields in certain assays compared to methanol, the efficiency of bioactive compound extraction should not be evaluated solely based on the quantity of target compounds obtained, but also by considering additional factors such as the stability of the extracts over storage time [57,58]. The literature reports indicate that the use of NaDESs not only enhances extraction efficiency, as observed in our study, but also contributes positively to the preservation of bioactive compounds under various storage conditions [6,10,59,60]. The stability of extracts throughout their shelf life is a critical parameter, particularly for industrial applications [61,62]. Conversely, although the reusability of the optimized NaDESs was not experimentally investigated in this study, it remains a crucial aspect that warrants thorough exploration in future research. Moreover, the recovery and reuse efficiency of NaDESs represent key variables within the Green Chemistry framework due to their direct implications for sustainability [63,64]. Although these aspects were not addressed in the present study, future research should prioritize their evaluation, in addition to assessing extract bioactivity, improving applicability, determining cost-effectiveness, and facilitating large-scale industrial adoption under a sustainable approach.

In contrast, methanol is recognized not only for its toxicity, but also for being flammable, corrosive, and non-biodegradable. Its synthesis poses environmental concerns due to the generation of by-products that contribute to air and water pollution, in addition to the health risks associated with its handling [65]. These considerations underscore the importance of adopting safer and more sustainable solvents such as NaDESs for industrial-scale applications in alignment with the principles of Green Chemistry.

### 3.6. Determination of the AGREE Index (Analytical Greenness Metric Approach)

**Figure 7:**
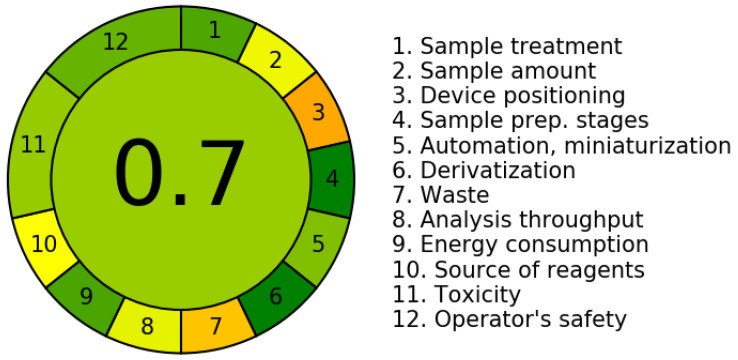
Green Chemistry Index for extraction and quantification of total phenolic compounds using natural deep eutectic solvents. Scores—sample treatment: 0.85; sample amount: 0.53; device positioning: 0.33; sample rep. stages: 1.00; automation, miniaturization: 0.75; derivatization: 1.0; waste: 0.39; analysis throughput: 0.55; energy consumption: 0.86; source of reagents: 0.50; toxicity: 0.71; operator’s safety: 0.8. Source: Agree—Analytical Greenness Calculator [19].

**Figure 8:**
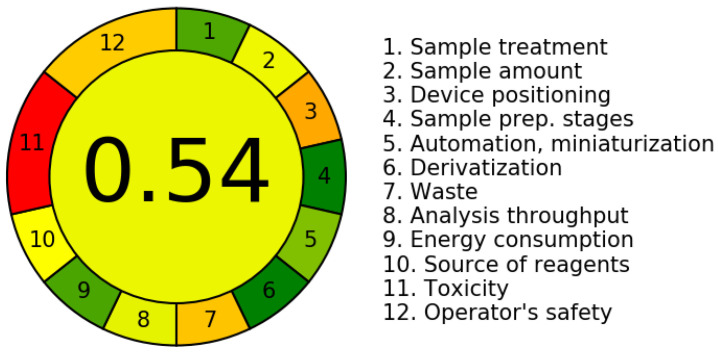
Green Chemistry Index for the extraction and quantification of total phenolic compounds using 70% methanol. Scores—sample treatment: 0.85; sample amount: 0.53; device positioning: 0.33; sample rep. stages: 1.00; automation, miniaturization: 0.75; derivatization: 1.0; waste: 0.39; analysis throughput: 0.55; energy consumption: 0.86; source of reagents: 0.50; toxicity: 0.0; operator’s safety: 0.4. Source: Agree—Analytical Greenness Calculator [19].

The sustainability evaluation of the extraction and quantification method for total soluble phenolic compounds based on the AGREE index highlighted that substituting methanol with NaDESs is a promising strategy in line with the Green Chemistry principles. The method employing NaDESs achieved an overall score of 0.7 (Figure 7), surpassing the score obtained with methanol (0.54) (Figure 8), reflecting notable improvements in environmental impact and operational safety.

The detailed evaluation of the criteria comprising the AGREE index revealed that although most parameters (such as sample treatment, quantity used, preparation steps, miniaturization, waste generation, analytical yield, energy consumption, and reagent origin) showed comparable scores for both the methods, the NADES-based methodology demonstrated clear advantages, specifically in criteria 11 (toxicity) and 12 (operator safety). This superior performance stems from the milder nature of the components in NADES solvents, which unlike traditional organic solvents like methanol are non-flammable, exhibit low-level toxicity, and present a reduced environmental impact risk [9].

However, despite the relative improvement, the index still reflects limitations such as the need for multiple extraction cycles to maximize yield. While this practice aligns with the exhaustive extraction strategy, recognized as a promising approach for the recovery of bioactive compounds, it inevitably leads to increased solvent consumption, energy demand, and analytical time, thereby negatively impacting the overall method score [66]. Consequently, strategies aimed at reducing the number of extraction cycles without compromising yield are essential to enhance the green profile of the process.

Moreover, the use of Folin–Ciocalteu reagent for total phenolic quantification remains a limitation due to its potential irritant properties, even though it is not classified as toxic. This underscores the need to replace it with safer alternatives that are better aligned with the principles of Green Chemistry [67]. In this regard, process miniaturization, coupled with the elimination of derivatization steps and the overall simplification of the methodology, is considered a promising approach, contributing to waste reduction and the improved reproducibility of results [68].

Furthermore, the other criteria assessed by the green index, such as energy consumption, waste generation, and operational efficiency, demonstrated comparable scores across the extraction systems evaluated. This observation suggests that although NaDESs represent a significant advancement in terms of safety and toxicological profiles, further optimization is required to minimize the overall environmental impact of the process. In particular, the implementation of successive extraction cycles to maximize phenolic compound recovery resulted in a proportional increase in solvent and energy usage, thereby reducing the final sustainability score. This outcome emphasizes the importance of rationalizing extraction steps to prevent operational overloads that may compromise the eco-efficiency of the method.

The assessment of the responses of the different food matrices under optimized extraction conditions revealed similar profiles between corn and buckwheat, as well as between red lentil and chickpea, suggesting a potential convergence in the solubilization mechanisms of TSPCs under the conditions applied. The application of experimental design and multivariate optimization techniques was crucial to ensure maximum extraction efficiency, while minimizing interference with critical environmental parameters.

### 3.7. Determination of the BAGI (Blue Applicability Grade Index)

**Figure 9:**
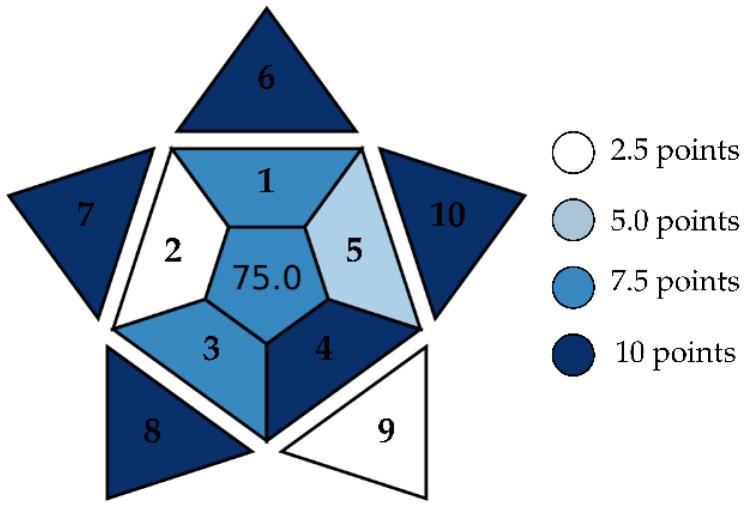
The practicality index of the method for the extraction and quantification of the total soluble phenolic compounds using NADESs and 70% methanol as extraction systems. Scores—(1) the type of analysis: quantitative; (2) the number of analytes that are simultaneously determined: single elements; (3) the analytical technique and required analytical instrumentation: simple instrumentation available in most labs; (4) the number of samples that can be simultaneously treated: >95; (5) sample preparation: miniaturized extraction sample preparation; (6) the number of samples that can be analyzed per hour: >10; (7) the type of reagent and material used in the analytical method: common commercially available reagents; (8) the requirement for preconcentration: no preconcentration required; (9) the automation degree: manual treatment and analysis; (10) the sample size: <100 µL. Source: BAG I-*Blue Applicability Grade Index*.

The graphical analysis of the BAGI (Figure 9) presents the practicality index of the method for the extraction and quantification of the total soluble phenolic compounds.

Additionally, the effects of thermal processing by extrusion on the stability of phenolic compounds were investigated, generally showing a significant reduction in the extractable levels post-processing. This reduction is likely attributable to the thermal degradation and structural transformation of free phenolic compounds, along with the reinforcement of interactions with the food matrix. However, an exception was noted for the biofortified orange-fleshed sweet potato, which exhibited an increase in TSPC content following extrusion, possibly due to the breakdown of phenolic macromolecular complexes or the release of previously bound conjugated forms.

The score of 75.0 assigned to the analytical protocol by the BAGI (Figure 9) highlights its technical reliability and the consistency of the criteria adopted for the extraction and quantification of total soluble phenolic compounds. The spectrophotometric technique based on the Folin–Ciocalteu reaction, combined with the use of natural deep eutectic solvents (NADESs), showed an excellent performance in aspects such as the type of analysis, the reagents used, operational simplicity, and its low sample requirement. These factors make the method accessible, practical, and environmentally sustainable, aligning with the principles of Green Chemistry [69,70].

In contrast, the BAGI identified relevant limitations regarding automation, simultaneous sample preparation, and multi-analyte determination, which reduce the scalability and analytical throughput of the method, critical points for large-scale studies or industrial applications. While spectrophotometry provides only a general estimate of the phenolic content, techniques such as HPLC would allow for the identification and quantification of specific compounds, such as gallic acid, rutin, and quercetin, with greater precision. However, these approaches would require higher instrumental complexity, longer preparation time, and increased operational costs [71].

## 4. Conclusions

This study demonstrated that the composition of the solvent exerts a significant influence on the extraction yield of TSPCs. Although 70% methanol resulted in higher quantitative yields, NaDESs were identified as non-toxic, biodegradable, and environmentally benign alternatives, fully aligned with the principles of Green Chemistry. The application of thermoplastic extrusion led to a reduction in the TSPC content in most samples, likely due to thermal degradation occurring during processing. In contrast, an increase in the TSPC content was observed in sweet potato following extrusion, presumably due to the release of phenolic compounds previously bound to macronutrients and to potential interference from intrinsic reducing sugars, which may have contributed to an overestimation of the phenolic content when using the Folin–Ciocalteu method.

Multivariate analyses confirmed that both the composition of the matrix and the processing conditions significantly influenced the TSPC levels. Although lower extraction yields were generally observed with the NaDESs, these solvents demonstrated a greater capacity to preserve phenolic compounds during storage, an attribute of considerable relevance for future studies and industrial applications. Furthermore, sustainability (AGREE) and practicality (BAGI) assessments indicated that NaDES-based extraction methods are effective and methodologically robust, accessible, safe, and environmentally favorable.

In conclusion, although additional research is required to evaluate the cost-effectiveness, scalability, and operational feasibility of NaDES-based extraction on an industrial scale, this approach represents a promising and sustainable alternative. It adheres to the foundational principles of Green Chemistry and holds significant potential for large-scale implementation in food and bioactive compound processing.

## Figures and Tables

**Table 1:** The coded and real levels of the constrained Simplex-Centroid Mixture Design used for the extraction of total phenolic compounds.

Trials	Coded Levels			Real Proportions (%)		
	x_1_	x_2_	x_3_	X_1_	X_2_	X_3_
1	1.0	0.0	0.0	98.0	1.0	1.0
2	0.0	1.0	0.0	1.0	98.0	1.0
3	0.0	0.0	1.0	1.0	1.0	98.0
4	0.5	0.5	0.0	49.5	49.5	1.0
5	0.5	0.0	0.5	49.5	1.0	49.5
6	0.0	0.5	0.5	1.0	49.5	49.5
7	0.6667	0.1667	0.1667	65.7	17.2	17.2
8	0.1667	0.6667	0.1667	17.2	65.7	17.2
9	0.1667	0.1667	0.6667	17.2	17.2	65.7
10	0.3333	0.3333	0.3333	33.3	33.3	33.3
11	0.3333	0.3333	0.3333	33.3	33.3	33.3
12	0.3333	0.3333	0.3333	33.3	33.3	33.3
13	0.3333	0.3333	0.3333	33.3	33.3	33.3

Where x_1_ and X_1_—3 M sorbitol; x_2_ and X_2_—60 mM citric acid; x_3_ and X_3_—300 mM glycine.

**Table 2:** Numerical optimization, experimental and predicted absorbance values (100 mg·mL^−1^), and relative deviation (%) for validation of mathematical models applied to extraction of total phenolic compounds from raw plant materials.

Raw Material	Independent Variables ^#^			Numerical Optimization and Validation			
	Sorbitol 3 M	Citric Acid 60 mM	Glycine 300 mM	Predicted Value	Experimental Value ^δ^	Relative Deviation	Desirability
Corn	0.9782	0.1180	0.1000	0.087	0.097 ± 0.009	10.62	0.9303
Buckwheat	0.9800	0.0100	0.0100	0.284	0.236 ± 0.019	−16.98	0.9272
Red lentil	0.0102	0.0100	0.9798	0.220	0.262 ± 0.012	18.81	0.9611
Chickpea	0.0100	0.0100	0.9800	0.290	0.317 ± 0.030	9.22	0.8598
Sweet potato	0.0100	0.9800	0.0100	0.235	0.213 ± 0.030	−9.07	0.4567
Sudan grass	0.9241	0.0658	0.0100	0.405	0.436 ± 0.065	7.49	0.9827

^#^ The optimal values for the independent variables are presented by coded levels; to convert them to actual values, interpret them as percentages. ^δ^ The experimental values are reported as the arithmetic mean of triplicate measurements ± standard deviation. The regression coefficients and ANOVA were assessed at a 10% significance level (*p* ≤ 0.10).

## Data Availability

All the data obtained in this study are available in this article.
